# Beneficial Effects of Ketogenic Diet on Phosphofructokinase Deficiency (Glycogen Storage Disease Type VII)

**DOI:** 10.3389/fneur.2020.00057

**Published:** 2020-02-04

**Authors:** Minna E. Similä, Mari Auranen, Päivi Liisa Piirilä

**Affiliations:** ^1^Clinical Nutrition Unit, Internal Medicine and Rehabilitation, Helsinki University Hospital, University of Helsinki, Helsinki, Finland; ^2^Clinical Neurosciences, Neurology, Helsinki University Hospital, University of Helsinki, Helsinki, Finland; ^3^Unit of Clinical Physiology, HUS Medical Imaging Center, Helsinki University Hospital, University of Helsinki, Helsinki, Finland

**Keywords:** Tarui disease, GSDVII, ketogenic diet, cardiopulmonary exercise capacity, lactate, ammonia, glykogen storage disease

## Abstract

**Background:** A deficiency of muscle phosphofructokinase (PFKM) causes a rare metabolic muscle disease, the Tarui disease (Glycogen storage disease type VII, GSD VII) characterized by exercise intolerance with myalgia due to an inability to use glucose as an energy resource. No medical treatment for GSD VII currently exists. The aim of this study was to determine whether a dietary intervention with excessive fat intake would benefit GSD VII.

**Patient and Methods:** A ketogenic diet (KD) intervention implemented as a modified Atkins diet was established for one patient with PFKM deficiency, with a low late lactate response and very high ammonia levels associated with exercise. We recorded the KD intervention for a total of 5 years with clinical and physiotherapeutic evaluations and regular laboratory parameters. Cardiopulmonary exercise testing, including breath gas analysis and venous lactate and ammonia measurements, was performed before KD and at 3, 8 months and 5 years after initiation of KD.

**Results:** During the 5 years on KD, the patient's muscle symptoms had alleviated and exercise tolerance had improved. In exercise testing, venous ammonia had normalized, the lactate profile remained similar, but oxygen uptake and mechanical efficiency had increased and parameters showing ventilation had improved.

**Conclusions:** This study is the first to show a long-term effect of KD in GSD VII with an alleviation of muscle symptoms, beneficial effects on breathing, and improvement in exercise performance and oxygen uptake. Based on these findings, KD can be recommended under medical and nutritional supervision for selected patients with GSD VII, although further research of this rare disease is warranted.

## Introduction

Tarui disease or muscle phosphofructokinase (PFKM) deficiency belongs to the glycogen storage diseases (GSD VII, OMIM#232800). It is a rare autosomal recessive disorder caused by mutations in the *PFKM* gene and characterized by exercise intolerance, muscle cramping, and myoglobinuria associated with compensated hemolysis and later nascent muscle weakness and mild myopathy (1, 2). Phosphofructokinase is the rate-limiting enzyme in the glycolytic pathway and catalyzes the phosphorylation of fructose 6-phosphate to fructose-1, 6-bisphosphate. In Tarui disease, typically the total lack of phosphofructokinase enzyme in muscle tissue causes the oxidative pathway from glucose to pyruvate to be blocked at this point of the glycolysis pathway, and glucose cannot be used in muscle energy metabolism normally. Therefore, the muscle tissue in Tarui disease must utilize alternative oxidative substrates other than glucose in energy metabolism. In McArdle's disease (GSD V, #232600), a condition resembling Tarui disease, increased fat oxidation during exercise has been reported (3). The importance of free fatty acids for muscle oxidative metabolism has also been revealed in Tarui disease, and patients with this disease have been shown to benefit from substrates available after overnight fasting or triglyceride infusion during aerobic exercise (4). Indeed, glucose would not have a favorable effect on their cardiorespiratory capacity, and glucose ingestion could even worsen the condition (4, 5). Patients with Tarui disease, unlike in McArdle's disease, do not show the second-wind phenomenon, an exercise-related increase in the capacity for muscle oxidative phosphorylation (6, 7). In both diseases, an excess of purine metabolites, e.g., uric acid, hypoxanthines, and ammonia, as an indicator of increased utilization of proteins has been found (8).

Currently, no specific treatment options exist for Tarui disease. Regular physiotherapy is important, although a benefit from slowly progressive exercise training has not been established (6, 7). However, a diet low in carbohydrates and high in fat and protein (ketogenic diet, KD) could hypothetically be beneficial for patients. In order to understand the benefit of KD in the long term we present the results of a careful 5 years follow-up with blood specimens, clinical visits, and cardiopulmonary exercise testing with blood gas, ammonia, and lactate examinations on a male patient suffering from Tarui disease (9, 10).

## Patient and Methods

### Patient

The patient is a 59 years-old man carrying a homozygous *PFKM* gene mutation, c.329G>A. The histological and genetic findings (9) and the analyses of the exercise lactate profile (10) have been published earlier. In childhood, he had normal motor development and normal exercise tolerability, but was not very keen on sports. As a young boy he could run up to 100 meters but around the age of 12 years he started to develop symptoms, including muscle weakness, and attacks of muscle pain, weakness, cramping, and vomiting, during extensive physical activity. He was never hospitalized due to the muscle symptoms or due to raise in creatine kinase (CK) values.

At age 59, there was mild muscle weakness in hip flexors and extensors and ankle flexors and extensors on the right side [MRC scale 4 out of 5 (11)], mild reticulocytosis without anemia, and normal EMG. No muscle atrophy was evident. Medication for high blood pressure had been recently introduced (amlodipin 5 mg and valsartan 160 mg once a day). The patient's weight was 71 kg and height 177 cm (BMI 23.0 kg/m^2^). He could walk with a slow pace about 10 km but walking on an incline was limited by muscle pain. He could not run, because of muscle cramping and feeling unwell, and could walk only one flight of stairs. He did regular hunting and hiking trips in the forest. He annually spent 4–5 days in Lapland with a group of friends hiking 15–20 km daily but he was always the last in the group and other members had to wait for him.

Ethical approval for the study was granted by the Medical Ethics Committee of Helsinki University Central Hospital, Finland. Informed consent was provided by the patient and the controls.

### Diet

The patient's diet was evaluated using a 3 days food record prior to KD initiation, and the evaluation was repeated 6 months after starting KD. Dietary intakes were calculated using the national food composition database of the National Institute for Health and Welfare, Finland (12). The KD was guided by the same dietitian (M.S.) throughout the study period. Nutrition and diet were assessed and counseled at clinic visits or by phone or email contacts several times during the initiation period and later at least yearly. Daily energy intake was planned to be at the same level as before the KD, the amount of carbohydrates was restricted to 10 g per day, and the consumption of fat and protein was encouraged, aiming at a ketogenic ratio of ~1:1. Consumption of unsaturated fats was recommended to avoid unfavorable changes in serum lipids. Multivitamin, additional vitamin D, and calcium supplementations were introduced. Temporal mitigation of carbohydrate restriction (using small or moderate amounts of e.g., rye bread) was allowed during the treatment when disadvantage of the diet was considered (strong increase of LDL-cholesterol).

### Laboratory Assessment

Laboratory parameters were followed up regularly; e.g., β-hydroxybutyrate, glucose, cholesterol, CK, and liver function were measured almost monthly during the first year and, after stabilization of the diet, twice during the second year and once a year thereafter, except for cholesterol values, which were measured more often. The laboratory results from the time points of exercise testing are presented in Table 1. In addition, concentrations of some vitamins (vitamins D and A), minerals (such as selenium and zinc), carnitine (total and free), prealbumin, and urine calcium and creatinine were measured discretionarily to optimize nutrient intakes. Blood β-hydroxybutyrate was also measured by the patient at home to ensure ketosis (target level 2.5–5), at the beginning every morning and evening, and later on demand.

**Table 1:** Laboratory parameters before ketogenic diet (KD) and during the 5 years follow-up at the time points of cardiopulmonary exercise testing.

**Parameter assayed reference range**	**Before KD**	**KD 3 months**	**KD 8 months**	**KD 5 years**
Plasma β-hydroxybutyrate (mmol/L) <0.2 mmol/L	0.22	1.30	0.59	0.4^**^
Plasma glucose (mmol/L) 4.0–6.0 mmol/L	5.8	5.2	5.3	5.6^**^
Serum insulin (mU/L) (2–20 mU/L)	4.1	1.6	1.2	3.5^**^
Plasma creatine kinase (U/L) 40–280 U/L	96	74	160	159
Plasma alkaline phosphatase (U/L) 35–105 U/L	55^*^	34	34	Not measured
Plasma alanine aminotransferase (U/L) 10–70 U/L	33	36	25	27
Plasma glutamyltransferase (U/L) 15–115 U/L	41^*^	18	24	18^**^
Plasma triglycerides (mmol/L) <2.0 mmol/L	2.39	0.64	0.59	0.67
Plasma cholesterol (mmol/L) <5.0 mmol/L	4.6	5.2	5.6	4.4
Plasma LDL^#^ cholesterol (mmol/L) <3.0 mmol/L	2.7	3.5	3.5	2.1
Plasma HDL^##^ cholesterol (mmol/L) >1.0 mmol/L	1.52	1.88	2.15	1.83

*Laboratory tests were studied once a month during the first year, three times in the second year, and annually in years 3–5*.

* *Measured during KD initiation*.

** *Measured 6 months before exercise test (values from the time of exercise test are missing)*.

# *Low density lipoprotein*.

## *High density lipoprotein*.

### Cardiopulmonary Exercise Test

Cardiopulmonary exercise test with breath gas analysis (spiroergometry) was performed as described earlier before KD and at 3, 8 months, and 5 years after start of KD (13). The test was started with a 40 W work load, with an increase of 40 W in 3 min increments (40 W/3 min). A maximal subjective level of at least 17/20 on the Borg scale was attained. The venous blood specimens were taken via a vacuum technique, and the analysis of lactate and ammonia specimens as well as the venous blood gases and electrolytes have been reported earlier (10). The results of four healthy men with a mean age of 48 (range 35–60) years and BMI 23.0 kg/m^2^ (SD 1.4) were selected from an earlier study (10) and served as a control. Younger men and women from the original controls were excluded.

### Statistical Methods

Spearman's correlation test was performed between the baseline and diet phases for the patient as well as for the controls.

## Results

### Diet and Laboratory Tests

Before KD, the calculated total energy intake was 2,660 kcal per day and consisted of 177 g of available carbohydrates (27 E%, percentage of total energy intake), 107 g of protein (16 E%) and 164 g of fats (55 E%). Six months after beginning KD, the calculated total energy intake was ~2,880 kcal per day and consisted of 229 g of fats (72 E%), 189 g of protein (26 E%) and 12 g of available carbohydrates (2 E%), with a ketogenic ratio of ~1:1.

The β-hydroxybutyrate concentrations measured in laboratory ranged between 0.4 and 2.9 mmol/L during the follow-up period mostly being below 2.5 mmol/L (Table 1). During periodical mitigation of carbohydrate restriction β-hydroxybutyrate concentration was lower. An increase was initially seen in cholesterol values, with the highest concentrations measured after 2 years on KD, total cholesterol 8.2 mmol/L, LDL-cholesterol 6.2 mmol/L and HDL-cholesterol 2.01 mmol/L. Ezetimibe 10 mg (Ezetimibe accord^®^) medication was started due to high cholesterol values. Finally, during medication, total cholesterol, and LDL-cholesterol were below baseline values and HDL-cholesterol was slightly increased at 5 years control. Plasma glucose and insulin values were within normal limits before and during KD and both decreased after beginning of the diet (Table 1).

Vitamin D concentration (serum 25-hydroxyvitamin D_3_ and D_2_) was at the beginning of dietary therapy below reference values, 32 nmol/L. After vitamin D supplementation, the concentration increased to 63 nmol/L.

### Clinical Examination

Already after 6 months' exposure to KD, the patient began to experience a subjective alleviation of muscle symptoms, manifesting as more rapid recovery and less muscle discomfort. He was able to increase daily exercise and could gradually spend extended periods of time hiking and hunting in the forest. However, he still often suffered from stiffness.

At 5 years, his muscle strength (MRC) was within the normal range (5 of 5) except for ankle extension forces (4 of 5). Deep tendon reflexes were present excluding Achilles. He felt that exercise tolerance had improved during KD and experienced less cramping and nausea during exercise than before KD. He could now walk longer without stopping and could ski 10–15 km/day at the same speed as his friends, which was not possible before KD. During KD he could participate in long hiking trips, moving at a similar speed as his peers, also not possible before KD. He felt that especially capability to walk on an incline was better than before KD.

The patient's weight had decreased from 71 to 58 kg (height 177 cm), with BMI falling from 22.7 to 18.5 kg/m^2^ in 5 years.

### Results of Cardiorespiratory Exercise Testing

The main results of the cardiorespiratory exercise testing during the follow-up at the time points of 3, 8 months and 5 years are presented in Table 2. The maximal working capacity was moderately reduced before KD, increasing slightly during KD, but it remained lower than the values of age-matched controls. The maximal oxygen uptake by body weight had a mild increase during KD and the mechanical efficiency (Wmax/VO_2_max) increased from 13.1 to 16.5% (normal value ≥20%).

**Table 2:** Results of cardiorespiratory exercise testing of the patient before and during KD.

	**Before KD**	**KD 3 months**	**KD 8 months**	**KD 5 years**	**Control subjects (*N* = 4) mean (SD)**
Heart rate maximum percentage of predicted (%)^*^	98.3	94.6	94.9	94.8	97.9 (4.6)
Borg subjective scale 6–20	19	19	17	20	18.3 (2.1)
RQ (V′CO_2_/V′O_2_) in maximal exercise	0.91	0.88	0.92	0.75	1.13 (0.05)
Wmax/3 min (maximal working capacity) (W)	93	90	99	102	285.2 (81)
V′O_2_ max (maximal oxygen uptake) (L/min)	2.045	1.838	2.159	1.778	3.79 (1.1)
V′O_2_/kg max (maximal oxygen uptake/weight) (ml/min/kg)	28.8	25.9	34.8	30.7	52.1 (12.1)
Wmax/V′O_2_ max (%)	13.1	11.1	14.1	16.5	21.7 (1.4)
Breathing frequency	68	61	56	46	43.0 (12.8)
Tidal volume, % of predicted	44.6	41.7	56.1	60.3	109.8 (24.8)
Fraction of end tidal CO_2_ (FetCO_2_) (%)	4.16	3.82	3.8	4.0	5.4 (0.51)

*RQ, respiratory quotient; V′CO_2_ is maximal CO_2_ production*.

* *205–0.5 × age*.

During KD a decrease occurred in the very high maximal breathing frequency in exercise, from 68 to 46/min and an increase in tidal volume from 44.6 to 60.3% of predicted value. However, slight hyperventilation was found, as assessed in slightly increased minute ventilation vs. CO_2_ production (VE/VCO_2_) and O_2_ consumption (VE/VO_2_) and also in slightly decreased FetCO_2_.

### Results of Venous Blood From Exercise Testing

**Figure 1:**
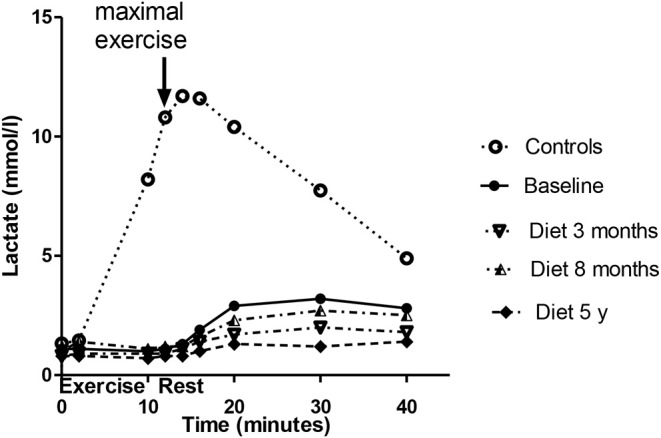
Lactate levels associated with exercise tests of the patient before diet and during the follow-up. The values of the control subjects without dietary intervention are also given for comparison. The baseline lactate data has been published earlier (10), the controls matched to the age and gender of the patient were obtained from the forementioned publication. A strong correlation existed between the patient's baseline lactate values and his diet curves at 3, 7 months, and 5 years, rho being 0.914 (*p* = 0.006), 0.885 (*p* = 0.015), and 0.90 (*p* = 0.001), respectively.

**Figure 2:**
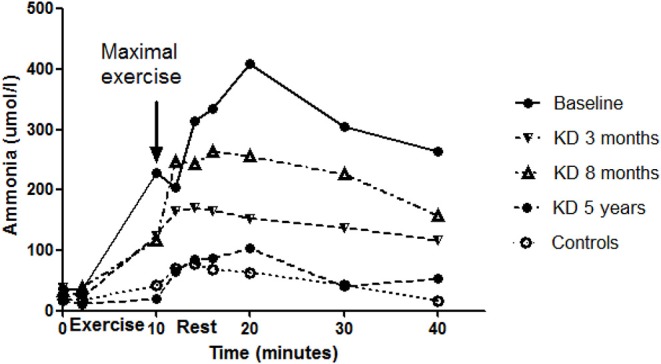
Ammonia levels associated with exercise tests of the patient before the diet and during the follow-up. Values of the control subjects without dietary intervention are also given for comparison. The baseline ammonia data has been published earlier (10), and the controls matched to the age and gender of the patient were obtained from the aforementioned publication. No correlations existed between the baseline curve of the patient and those of the controls or the diet curves of the patient.

**Figure 3:**
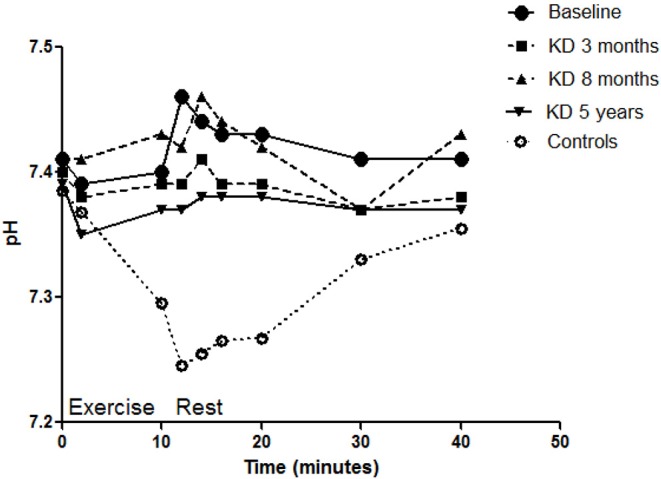
pH levels associated with exercise tests of the patient before the diet and during the follow-up. Values of the control subjects without dietary intervention are also given for comparison. The baseline ammonia curve has been published earlier (10), and the controls matched to the age and gender of the patient were obtained from the aforementioned publication. A high negative correlation existed between the patient's baseline curve and mean diet curve (rho = −0.826, *p* = 0.006).

The venous lactate level at rest remained at pre-KD level (1.1 mmol/L) in the follow-up (0.8 mmol/L) (Figure 1). The ammonia level at rest decreased from 36 to 27 μmol/L. The maximal ammonia level (measured 4–10 min after exercise) was 409 μmol/L before KD (10) and was reduced at 3 months to half of the pre-KD level. However, a new increase was seen at 8 months (after the periodical mitigations of carbohydrate restriction), which at 5 years had returned to normal level, 111 μmol/L (Figure 2). Before KD, metabolic alkalosis was seen after exercise (pH 7.46 and BE +4.2 mmol/L at 2 min after exercise), but at 5 years the pH value after exercise was within normal limits (pH 7.38 and BE + 2.6 mmol/L) (Figure 3).

## Discussion

During the 5 years follow-up of KD the patient remained clinically stable with subjective alleviation of muscle pain symptoms and better exercise tolerance. In cardiopulmonary exercise testing, working capacity and mechanical efficiency had increased. In venous blood, lactate levels had decreased from the low pre-KD levels, and the very high ammonia levels associated with exercise testing detected in the measurements before KD had decreased to normal. KD had a beneficial impact also on respiratory parameters during exercise, reflected as lowering of breathing frequency and the ventilatory equivalent for O_2_ (V′E/O_2_) and as increasing tidal volume, suggesting diminished hyperventilation. However, the end tidal CO_2_ level (FetCO_2_) remained rather low, indicating permanent hyperventilation tendency.

Increasing evidence is emerging for a benefit of dietary therapy, especially in metabolic muscle diseases (14), but also in dystrophic disorders (15). However, among GSDs specific enzyme replacement therapy exists only in Pompe's disease (GSD II) (16). In McArdle's disease (GSD V), some data suggest a benefit of KD in relieving symptoms (17). In one child with PFKM deficiency presenting with congenital arthrogryposis and severe myopathy, KD starting at age 4 months alleviated clinical symptoms, enhanced motor skill development, and improved muscle strength (18). Before KD, our patient's diet fat content (55 E%) was relatively high and carbohydrate content (27 E%) low compared with the typical diet of Finnish men (fat 36 E%, carbohydrates 42 E%) (12), possibly attenuating our findings on the beneficial effects of KD. The patient's original diet was evaluated for the first time before the initiation of KD, but had remained similar for years.

KD was fairly well-tolerated by the patient. About 7 months after adopting KD, high LDL-cholesterol level was measured (4.9 mmol/L). Since cardiovascular disease exists among the patient's immediate family, the disadvantage of the KD for the patient was reconsidered, and the restriction of carbohydrates was mitigated (e.g., rye bread was allowed), which led to a decrease in ketosis. Based on the patient's subjective experience of lowered exercise capability after the increase of carbohydrates, however, the KD was again tightened. With rising LDL-cholesterol values (up to 6.2 mmol/L) cholesterol-lowering medication was started after 3 years of KD. The LDL-cholesterol level was at the 5 years control lower than before KD (2.1 vs. 2.7 mmol/L). During follow-up, no abnormalities were detected in the liver or kidney functions. The patient's weight decreased by 13 kg during KD. He was satisfied with the weight decrease, although it was not the original aim of the treatment. Interestingly, the reported energy intake during KD was greater than before KD. This may be due to the better working capacity, leading to increased physical activity during the diet. However, it is also possible that the difference between these calculated intakes is explained by normal day-to-day variation in food intake since the food record periods were short (3 days).

The patient's maximal oxygen uptake (L/min) actually decreased in the follow-up, but relative to weight oxygen uptake mildly increased from the pre-KD values [from 91 to 103% of predicted values (19)]. The benefit of KD was more clearly seen in the mild increase in maximal working capacity, which rose from 56 to 68 W, with a simultaneous decrease in maximal heart rate. The mechanical efficiency, which is the maximal working power during the exercise test relative to the simultaneous maximal oxygen consumption (uptake), also increased during KD, reflecting improved exercise performance. However, at 5 years on KD, his exercise capacity remained at 68.1% of healthy controls' values (Table 2). The decrease in the respiratory quotient (RQ, relation of CO_2_ production to O_2_ consumption) at 5 years may more be associated with decreased glucose oxidation and increased fat oxidation (20) than with submaximal effort. This would be in line with former findings of inverse connection between free fatty acid concentration and oxidation with insulin concentration (21) since insulin adequately decreased after beginning of the KD.

Before KD, a very low lactate level was found in the cardiopulmonary exercise test, with a delayed increase occurring 10–20 min after the exercise (10). During KD the lactate curve associated with exercise testing had a similar shape as before the diet, but was situated lower, which is explained by less ingested carbohydrates. The level of ammonia increased to very high levels in exercise testing before KD compared with control subjects (Figure 2) as well as with earlier data on exercise tests in healthy subjects (22). High levels of ammonia might cause the patient's exercise fatigue and short-term neurological discomfort, as reported also in sport medicine (23, 24), being one explanation for the increased ventilation, especially during exercise. With KD, the level of ammonia during exercise was drastically decreased, attaining normal levels. In line with our finding, elevated ammonia has earlier been reported in Tarui disease during exercise testing (8).

A common route to catabolize proteins and nucleotides is deamination of adenosine monophosphate (AMP) to inosine monophosphate (IMP) (23–25) which generates ammonia. In Tarui disease, the increase of ammonia during exercise has in some reports been explained by impaired cellular adenosine diphosphate (ADP) phosphorylation and its increased degradation to AMP (6). The results of our earlier study (10) suggested that in Tarui disease, glucose passing the normal glycolytic route could be used in other anabolic biosynthetic reactions leading to increase of ammonia (26–28).

Just before the exercise test at 8 months the patient returned to ketosis, and in the exercise test, a new increase in ammonia was detected. This suggests that even a short period of more carbohydrates may increase the ammonia level during the exercise test (Figure 2) highlighting the importance of carbohydrates in the high ammonia levels associated with Tarui disease.

Since convincing evidence between higher whole-grain consumption, which is a substantial contributor to carbohydrate intake, and lower risk of coronary heart disease, type 2 diabetes, colorectal cancer and all-cause mortality, is growing (29), extreme restriction of carbohydrates has to be well-founded. The risk factors of chronic diseases must be considered during long-term KD as was done in our case, with cholesterol-lowering medication started due to the increase in LDL-cholesterol levels. The patient felt that his exercise capability was decreased in conjunction with increased carbohydrate intake and wanted to continue the strict KD with subsequent initiation of cholesterol medication.

During KD associated with the exercise test, pH and base excess values remained rather high, indicating that no metabolic acidosis had developed. Carbohydrate intake restriction and ingested fat and proteins produce acidic ketones, which have a tendency to advance to metabolic acidosis; however, this was not seen here. This is probably explained that there still would have been release of alkaline ammonia from protein catabolism, although less than before KD.

In our previous study (10), ventilation during exercise of the patient was markedly increased before KD, which may arise from lower exercise tolerance and increased ammonia levels during and after exercise causing neural discomfort. In the present study, better cardiopulmonary condition and lower ammonia production possibly lowered the ventilation. Correspondingly, in an earlier study of Tarui disease, exercise tests during triglyceride infusion were associated with decreases in ventilation and respiratory exchange ratio and increases in work intensity and oxygen uptake (4).

The main strength of this study is that KD was carefully implemented and followed in a very cooperative patient. Laboratory examinations were performed continuously, the follow-up intervals increasing as the patient achieved a balance with KD. Also the cardiorespiratory exercise testing was first performed with shorter intervals, and the last test at 5 years to obtain data on long-term KD treatment. A study limitation is that we had only one patient, and further studies are needed.

In conclusion, KD intervention in our patient with Tarui disease seemed to have a beneficial effect, as measured by the patient's subjective condition with diminished muscle symptoms and increases in exercise capacity and oxygen uptake, which also had a favorable effect on the patient's ventilation. Cholesterol values increased on the diet, but with statin medication the levels were kept under control. Our results thus encourage the implementation of KD in patients with Tarui disease as the risk factors of chronic diseases are considered.

## Data Availability Statement

The datasets for this article are not publicly available because of legislation.

## Ethics Statement

The studies involving human participants were reviewed and approved by Medical Ethics Committee of Helsinki and Uusimaa, 199/13/03/01/11. The patients/participants provided their written informed consent to participate in this study.

## Author Contributions

PP: spiroergometric testing of patient, interpretation and analysis of data, and drafting of the article. MA: treatment of the patient with Tarui disease, data analysis, and drafting of the article. MS: treatment of the patient, the dietary planning and follow-up, data analysis, and drafting of the article.

### Conflict of Interest

The authors declare that the research was conducted in the absence of any commercial or financial relationships that could be construed as a potential conflict of interest.
